# Artificial intelligence-driven screening, early diagnosis, and treatment strategies for cervical cancer: an overview

**DOI:** 10.1186/s13027-025-00716-5

**Published:** 2025-11-28

**Authors:** Mayuri Pawar, Samradni Pingale, Kavita Kadam, Ashwini Jadhav, Ruchika Kaul-Ghanekar

**Affiliations:** 1 Symbiosis School of Biological Sciences (SSBS), Symbiosis International (Deemed University) (SIU), Lavale Gram Village, Mulshi, Pune, Maharashtra 412115 India; 2 Symbiosis Centre for Research and Innovation (SCRI), Symbiosis International (Deemed University) (SIU), Lavale Gram Village, Mulshi, Pune, Maharashtra 412115 India

**Keywords:** Artificial intelligence, AI algorithms, Cervical cancer, Vaginal microbiome, Metabolome

## Abstract

Cervical cancer (CC) is a significant global health issue, particularly in low-income countries. Early detection and successful treatment techniques are critical for decreasing death rates. Artificial intelligence (AI) has emerged as one of the major transformational tools in cancer prediction, improved screening, diagnosis, and therapy. This comprehensive narrative review describes applications of AI in diagnosis, biomarker prediction, development of drugs, and tailored treatment for cervical cancer. Various studies reporting the use of AI-driven imaging methods, multi-omics data analysis, and deep learning algorithms were evaluated for their influence on enhancing CC treatment. AI-powered screening approaches, such as automated Pap (Papanicolaou) smear analysis and colposcopy interpretation, outperformed traditional techniques in terms of accuracy and efficiency. Machine learning algorithms helped in identifying crucial biomarkers, such as microbial and metabolic fingerprints, which improved early diagnosis. AI-assisted drug development has resulted in the identification of new therapeutic targets and improved chemotherapy regimens. Personalized medical techniques, based on AI-driven multi-omics data analysis have helped to increase patient outcomes. However, issues including dataset constraints, clinical validation, and ethical considerations must be resolved before the broad adoption of AI-based diagnosis and therapy. Future research should focus on improving AI models and incorporating them into clinical practice to improve CC management.

## Introduction

Cervical cancer (CC) ranks as the fourth most prevalent type of cancer among women globally and the second most common cancer among women in India [1]. CC is expected to kill 342,000 people in underdeveloped nations by 2020 [2]. Limited access to screening programs may be the primary cause of increased occurrence and fatality rates. Cervical dysplasia, commonly referred to as cervical intraepithelial neoplasia (CIN), has now been classified into 2 major categories, named as low-grade squamous intraepithelial lesion (LSIL) and high-grade squamous intraepithelial lesion (HSIL). The previously recognized CIN1, CIN2, and CIN3 are now categorized into LSIL and HSIL, where CIN 2/3 belongs to the HSIL category [3]. Cervical dysplasia, commonly referred to as cervical intraepithelial neoplasia (CIN), is classified into four categories: invasive cervical cancer (ICC), squamous cell carcinoma in situ (CIN 3), mild (CIN 1), and moderate (CIN 2). CIN can be identified before it develops into an ICC. Therefore, if precancerous signs are identified early, it will help in timely therapy and thus CC can be prevented at an early stage. The World Health Organization (WHO) recommends Human Papillomavirus (HPV) DNA or Visual Examination with Acetic Acid (VIA) testing for CC in low-resource countries [4]. In developed countries, VIA is performed with a colposcope; however, such facilities are scarce in low-income countries because of limited financial resources, poor infrastructure, and lack of healthcare professionals. Moreover, several studies have reported variations in the sensitivity of VIA and conventional colposcopy. Another widely employed cervical screening method is the Pap (Papanicolaou) smear. This requires manual inspection of the entire slide using a microscope. Visual analysis of Pap images requires skilled manpower, making it costly, labour-intensive, and error-prone. Various automatic Pap smear screening systems, such as the AutoPap 300, PapNet, Cytyc, and ThinPrep, have certain limitations that lead to poor accuracy [5]. Liquid-based cytology (LBC) is another method for processing cervical lesions, but there is no significant difference in detecting cervical dysplasia between the LBC and Pap smear methods [6]. Therefore, new and innovative point-of-care (POC) screening and diagnostic solutions are needed. Significant progress has been made in computerized microscopy diagnostics at POC, but their clinical usage has been moderate. With innovations in digitalization and imaging, it has become easy to classify various diseases such as cancer, cardiac disorders, psychiatric or neurological issues, and kidney and retinal diseases [7].

Artificial intelligence (AI) has recently gained worldwide importance because through its intricate algorithms, it can recognize and process images, and classify CC and precancerous tissue with greater accuracy [8]. Different AI technologies have been reported in CC segmentation and detection through HPV testing, cervical cytology, colposcopy, and Magnetic Resonance Imaging (MRI) [9, 10]. However, AI implementation in biomarker prediction, novel drug therapy, and precision medicine for overall improvement of CC management has not been reviewed in these articles. Here, we have attempted to deliver an overview of recently published studies on AI-based identification of biomarkers in CC, drug discovery and repurposing, development of new therapies to reduce side effects, and role of AI in developing precision medicine for CC.

## Methodology

This article provides a thorough overview of AI applications in CC diagnosis and prediction. Researchers reviewed both conventional and novel techniques, underlining the role of AI in CC screening and predictions available in the field. The evaluation has covered data from January 2014 to February 2025, including the most recent published investigations. The search approach included an extensive review of internet databases such as SCOPUS, Web of Science, and PubMed. Specific search phrases such as ‘cervical cancer’, ‘diagnosis’, ‘screening, ‘radiology’, and ‘machine learning (ML)’, ‘Deep learning (DL)’ were used to ensure a thorough evaluation. This study examined various literature, including original research papers, review studies, systematic reviews, and meta-analyses. The emphasis was on understanding the positive impact of AI applications on CC diagnosis and prediction compared to the standard approaches. Articles in languages other than English were unfortunately excluded owing to linguistic limitations. We have included total 46 articles in this review. This article is an excellent resource for understanding the critical role of AI in CC research, including detection approaches and novel advances in medication and drug delivery. The inclusion of a varied set of papers as well as a rigorous search procedure has added to the review’s comprehensiveness. 

### Inclusion criteria

We have included the articles published in peer-reviewed conference papers and journals that were written in English language from January 2014 to February 2025. Only those articles were selected that emphasized the use of AI in cervical cancer.

### Exclusion criteria

Those articles were excluded from the study that did not specify the use of AI in cervical cancer, were published before January 2014 and after February 2025, and were available in languages other than English.

## Artificial intelligence in CC

### Conventional methods for CC diagnosis

**Fig. 1:**
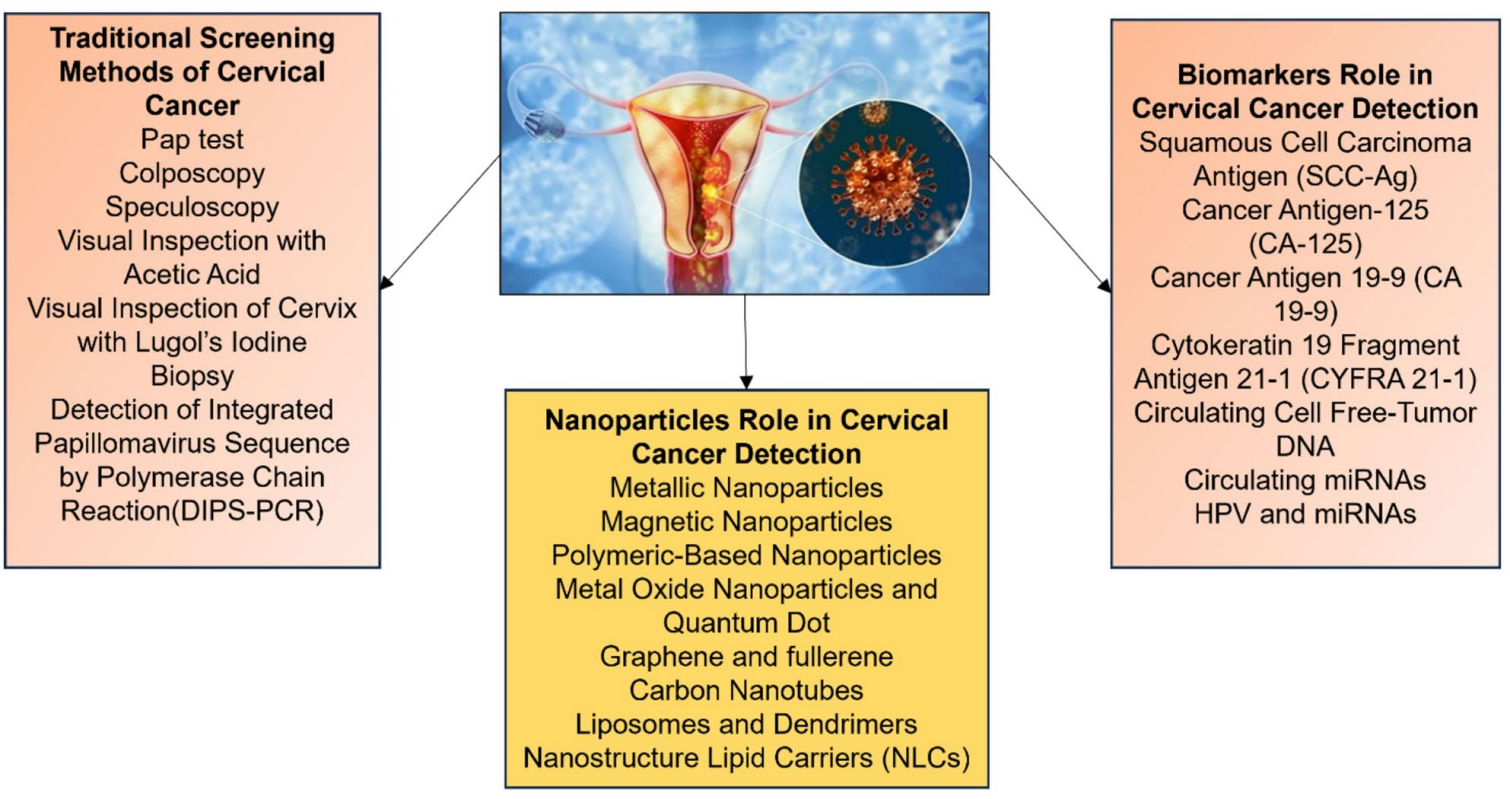
Applications of traditional screening methods, nanoparticles and biomarkers for the detection of cervical cancer (CC)

Various traditional methods are used for the detection of CC (Fig. 1) which include pap test, colposcopy, speculoscopy [11, 12], visual inspection with acetic acid, visual inspection of the cervix with Lugol’s iodine, biopsy, and detection of integrated papillomavirus sequence by polymerase chain reaction (DIPS-PCR). Among these strategies, the Pap test is the pillar of screening that can reduce mortality rate of CC. But the manual investigation of tests is laborious, relatively less sensitive and specific. Moreover, there is a high chance of missing the malignant cells during manual screening, which may jeopardize the ongoing treatment strategy. Although semi-automated frameworks for Pap test screening have been created, they are associated with bulky, costly research facility hardware and are not appropriate for use at the POC or in resource-limited settings. Among the other screening methods, different nanoparticles such as metallic nanoparticles, magnetic nanoparticles, polymeric-based nanoparticles, metal oxide nanoparticles, quantum dots, etc. have been used for the enhanced detection of CC by incorporating them into biosensors or various devices. Expression levels of different biomarkers such as squamous cell carcinoma antigen (SCC-Ag), cancer antigen-125 (CA-125), cancer antigen 19 − 9 (CA 19 − 9), cytokeratin 19 fragment antigen 21 − 1 (CYFRA 21 − 1), circulating cell-free tumor DNA, and circulating microRNAs (miRNA) HPV and miRNAs, could help to predict CC (Fig. 1).

## Applications of AI in CC

**Fig. 2:**
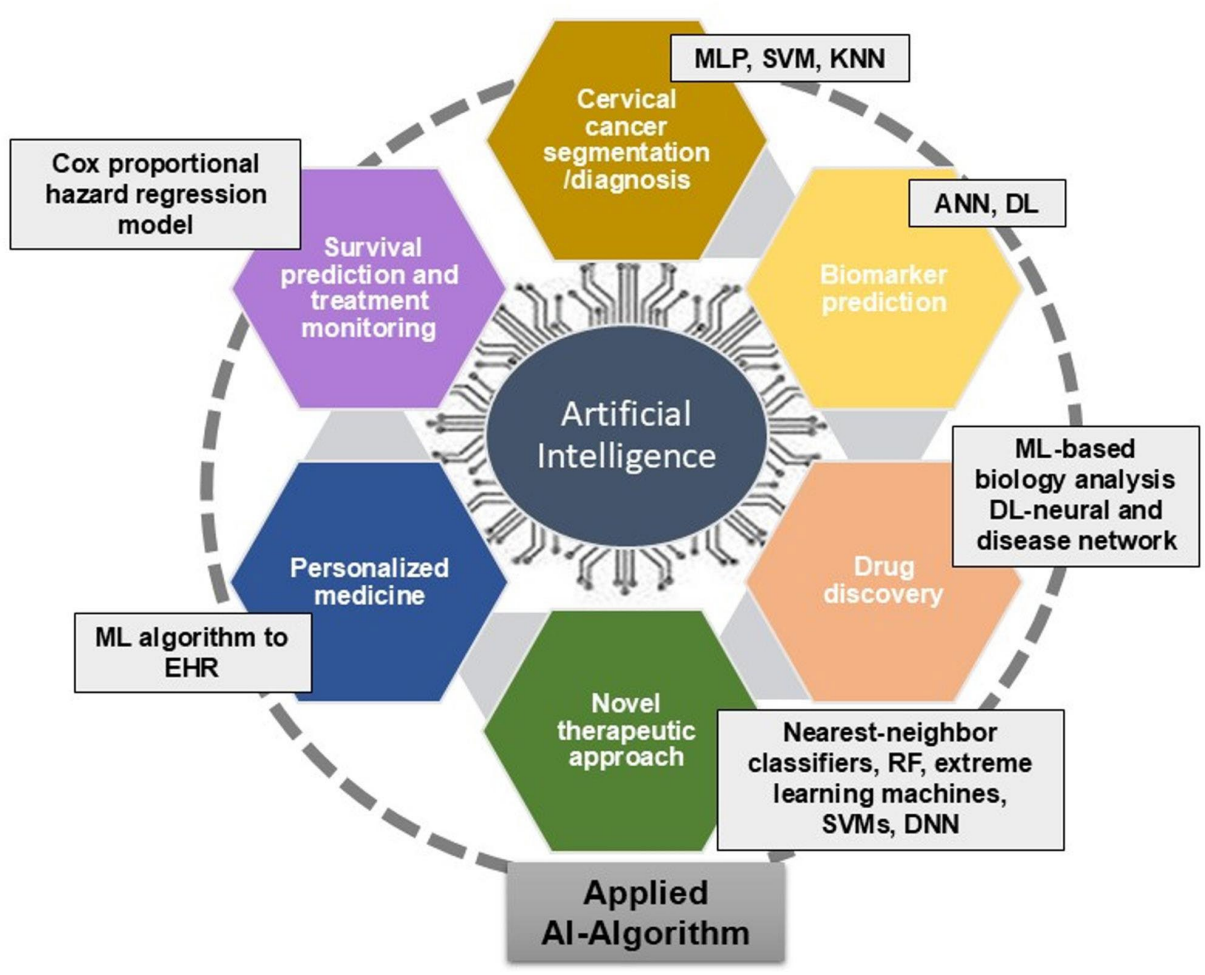
Applications of different AI-based algorithms for diagnosis, biomarker prediction, drug discovery, novel therapy, personalized medicine, survival prediction and treatment monitoring in CC

AI technologies, such as ML, can significantly improve the current CC screening [13–17]. AI can accelerate the discovery of novel drug molecules and the synthesis of more promising drug molecules. In doing so, it can substantially speed up the development of anticancer drugs and is expected to have a significant role in human cancer research and treatment, especially in CC. Recently, the application of AI-based algorithms has made a unique contribution to the screening, diagnosis, novel drug discovery, and treatment of CC (Fig. 2). AI algorithms such as a multi-layer perceptron (MLP), support vector machine (SVM), and K-nearest neighbor (KNN) are capable of detecting CC; and use a similar kind of metrics for evaluation. DL, a type of Artificial neural network (ANN), and other ANNs can predict biomarkers. ML-based biology analysis and DL-based neural and disease networks are used for drug discovery. Nearest-neighbor classifiers, RF, extreme learning machines, SVMs, and deep neural networks (DNNs) are useful in seeking novel therapeutic approaches. ML can be applied to electronic health records (EHR) for predicting CC and personalized cancer care. Models like cox proportional hazard regression model are beneficial for survival predictions and treatment monitoring.

### AI for CC screening and diagnosis

Researchers have been exploring the use of AI in analyzing medical images, such as Pap smears, colposcopy images, and histopathological slides, to improve the early detection and accurate diagnosis of CC. Various studies have emphasized the use of pap smear images for the detection of CC [18–20]. For instance, the Graph cut method applied on pap smear images requires less data and less time to train, shows more accuracy than the other methods [18], but it is limited to binary problems and is more sensitive to parameters and noise than a convolutional neural network (CNN). Some studies [19, 20] have used different CNN models, such as Random Forest classifier, ResNet-34, and EfficientNet-B3 models DenseNet-201, which usually require a large, high-quality database to evaluate; it can learn intricate patterns automatically. However, it is not useful for rare CC lesions, as the requirement for a large quantity of data also requires complex training.

Different AI models such as Markov model [21], Logistic regression, multi-layer perceptron (MLP), support vector machine (SVM), K-nearest neighbors (KNN), and several naïve Bayes [22] AI-assisted models [23, 24] have been reported for screening of CC with the help of LBC (Table 1). These models have increased specificity and sensitivity and along with LBC can help health professionals and technicians to enhance the accuracy of results and reduce errors. A study conducted by Kim et al. (2023) [25] used colposcopic images to train an ML algorithm to differentiate between HSIL and LSIL. U-Net model and DL classifier model have been reported to analyze colposcopic images of CC lesions before and after acetic acid wash [26, 27]. Mascarenhas et al. (2024) studied a CNN model constructed using ResNet model, which enables skip connections that allow information to bypass layers, is easy to train and improves convergence and performance [28].

As mentioned earlier, VIA is a low-cost method to detect CC; however, it requires a substantial amount of training and quality assurance. An enhanced visual assessment (EVA) and automated visual evaluation (AVE) AI tool have been developed that can be operated on smartphones to analyse the captured images of the cervix for identifying pre-cancerous lesions [29, 30]. AVE with AI can accurately detect and classify complex and subtle defects that traditional systems fail to recognize. It enables self-learning, is adaptable, sustainable and economically viable. A recent publication proposed the implementation of an algorithm termed The Hunger Games search optimization (HGSO) technique, for the training of an artificial neural network (specifically, MLP) aimed to detect CC with a minimal error rate [31]. The HGSO operates without relying on gradients and utilizes a population-based approach; however, it has inadequate diversity, premature convergence, and susceptibility to local optima. Similarly, another investigation employed MLP to differentiate between healthy samples and those exhibiting cervical lesions by integrating the Bayesian regulation algorithm [32]. Additionally, a Swin transformer, in conjunction with genetic algorithm feature selection and a random forest classifier, was utilized to categorize images derived from the Pap smear for the assessment of CC. Swin transformer is efficient on high-resolution images, reduces computational complexity, and delivers robust results. It may require large computational resources and memory, and may underperform CNN when highly localized features are involved [33]. Another analysis involved the examination of images obtained from pap smear using an automated cervical precancerous lesion classification system, which incorporated quantum invasive weed optimization alongside deep learning, demonstrating superior accuracy compared to the pre-existing algorithms [34]. An ensemble classifier that integrates random forest, Gaussian naïve Bayes, decision tree, SVM, and SHapley additive exPlanation as a visualizer was developed to facilitate the precise diagnosis of CC [35]. Furthermore, another study underscored the application of p16-positive regions on immunohistochemistry (IHC) samples collected from 111 patients to predict the presence of HSIL. For this purpose, the AI models Swin-B and Res-Net were employed to classify cervical lesions into the HSIL category [36].

Another study demonstrated the application of an enhanced ResNet-50 along with LIME (Local Interpretable Model-agnostic Explanations) to achieve improved classification of cervical images into malignant and non-malignant states [37]. In an one more study, a specific deep learning framework was used to develop a robust model for the detection of cervical malignancies through the segmentation of cervical regions and acetowhite lesions collected from the patients [38]. A reinforcement learning cancer network (RL-CancerNet), alongside a Vision Transformer integrated with Particle Swarm Optimization and Support Vector Machine (ViT-PSO-SVM) was developed and examined to enhance the diagnostic accuracy for CC. For this initiative, CC images were sourced from various publicly accessible datasets, including SipaKMed and Herlev, among others. ViT has a self-attention mechanism, which is highly data dependent [39, 40]. Moreover, an AI model was designed to analyze digital images obtained from thecervixes of 9,542 women to identify treatable precancerous lesions. The analyzed model yielded reproducible outcomes and was capable of distinguishing between precancerous lesions and healthy tissue regions within the digital images of the cervix [41]. In another study, cervical images obtained from pap smear test, VIA, histopathology, LBC, etc. were analyzed using various AI-based algorithms to differentiate between healthy, pre-cancerous, and cancerous lesions [42–46].

**Table 1:** AI-based algorithm models used for screening or diagnosis of CC

Model	Study	Model performance	Study outcome	References
Improved Boykov graph cut-based conditional random fields and superpixel-imposed semantic segmentation technique (IBGCCRF-SPSST)	Uses pap smear images for screening of CC	IBGCCRF-SPSST is a graph-based method that provides information about nuclei, their shape, size, and spatial distribution, it has 99.78% accuracy in a mean processing time of 2.18 s, which helps manage complex images.	The method can be useful to assist cytologists in the more precise detection of CC.	[18]
Convolutional Neural Networks (CNN)	Data of pap smear whole slide images from annotated Herlev and SIPaKMeD databases.	Binary, multi-classification system for the detection of CC that shows 99.70% accuracy, 99.70% precision, 99.72% recall, and 99.63% specificity.	A multi-classification system can provide another opinion than that of an expert analyst.	[19]
13 different pre-trained CNN models	Used publicly. available pap smear image data		DenseNet-201 performed best in terms of accuracy of 87.02% and performance	[20]
Markov model	The cohort study analyzed the cost-effectiveness of screening methods	The cost-effectiveness of the AI-assisted LBC technique is greatly impacted by its sensitivity and specificity.	Economically feasible to use an AI-driven LBC strategy once every five years rather than HPV DNA testing.	[21]
Logistic regression, MLP, SVM, KNN, and several naive Bayes	CC data from 859 female patients collected from public domain, each sample with Hinselmann, Schiller, biopsy, and cytology outputs	Area Under Curve (AUC) over 0.99	Class balancing techniques applied to achieve high performance in CC classification	[22]
Triaged using an AI-enabled liquid-based cytology, human cytologists, and HPV16/18 genotyping	Cytology slides	Sensitivity 86.49% vs. 83.78%, *P* = 0.744)	In areas with a shortage of skilled cytologists, AI-LBC may be especially helpful.	[23]
Online artificial intelligence for cytological assessment	Use of an online artificial intelligence system for screening of CC	The specificity of AI is higher than experienced cytopathologists	Artificial intelligence-based screening in Hubei provided a cheap, accessible, and effective way for screening CC	[24]
ML algorithms	Randomized, Double-blind controlled trial (7457 images from colposcopy)	AI system enhanced sensitivity (89.18%) and specificity (96.68%)	An AI-assisted system enabled easy distinction between low and high-grade lesions and also found the pathologic region	[25]
U-Net	Used colposcopic images taken before and after the acetic acid solution wash	Before acetic acid wash accuracy = 0.894, precision = 0.837, F1 score = 0.834. After acetic acid wash accuracy = 0.894, precision = 0.823, F1 score = 0.823	The model shows more accuracy for images that were taken before the acetic acid wash.	[26]
A deep learning classifier model	Colposcopic images after 56 s of acetic acid wash	Model showed sensitivity = 63%, specificity = 74%	This model has shown consistency through all metrics, adapts various imaging tools to classify HSIL and LSIL.	[27]
A CNN model constructed using ResNet model	A retrospective study of colposcopic images	The model had 99.7% sensitivity, 98.6% specificity.	The model performed well while distinguishing between HSIL and LSIL	[28]
MobileODT EVA system	Cross-sectional observational study, 2050 women participated in this study		From the study, it was found that EVA is more sensitive to HSIL and cancer than LSIL	[29]
AVE algorithm	8204 women participated in this study between the ages of 25–55. Visual inspection with acetic acid		It was helpful to detect CC in a poor-income country without any expensive instruments	[30]
Cascaded Multilayer Perceptron (c- MLP) model was trained using Bayesian Regulation algorithm	917 Pap smear images from Herlev open source dataset were considered in the study of which 649 images were used for training the model while 268 were used for testing to distinguish between normal and cancerous cervical cells	c-MLP had an accuracy of 97.63%	c-MLP model outperforms four traditional ML classifiers namely SVM, RFC, XCBoost, and Decision Tree.	[32]
Novel Swin- genetic algorithm feature selection- random forest (Swin-GA-RF)	3644 pap smear images from SIPaKMeD1 The dataset was used of which 70% training was used for 20% for validation and 10% for testing	Swin-GA-RF model had the highest accuracy in binary (99.012%) and five class classification (98.809%) when utilizing Adam optimizer	It has a higher accuracy than other Swin transformer and CNN pre-trained models.	[33]
AI model was built using OpenMMLab series having Swin-B as a Backbone	6171 patches from 111 patients were used of which 80% were used for training while 20% for testing	A novel AI model has high accuracy of 84.5%		[36]
SIPaKMeD pap-smear imagine dataset and Deep Learning classification algorithms to screen for CC using ResNet-50	-	The model has an accuracy of 91.04%	The incorporation of LIME Explainability aims to enhance the interpretability and transparency of the diagnostic process.	[37]
Deep Learning	Colposcope images	Precision, 0.7387 ± 0.1541; accuracy, 0.9291; specificity, 0.9589 ± 0.0131	-	[38]
Reinforcement Learning Cancer Network, “RL-CancerNet	Publicly available datasets, SipaKMeD and Herlev	Model performed with accuracy, 99.85%; precision, 99.80%; recall, 99.97%; sensitivity, 99.55%, and specificity, 99.35%	The proposed model suggests that it could be highly effective for early detection and treatment planning in clinical settings	[39]
Vision Transformers (ViT), AI-based diagnostic models to predict CC	Cervical cell image datasets such as SipakMed and Herlev	99.112% accuracy and 99.113% F1-score for SipakMed	The study demonstrated the feasibility and efficacy of the proposed model that could provide a robust, reliable, accurate, and non-invasive diagnostic tool for CC	[40]
Deep neural networks for cervical screening	Digital images of the cervix	Strong quadratic weighted kappa (QWK) of 0.86	Designed reproducible and a clinically translatable deep-learning model for cervical screening	[41]

Among the AI-based diagnostic models involving imaging datasets, Convolutional Neural Networks (CNN) model was observed to have higher (99.7%) sensitivity and (99.63%) specificity. On the other hand, RL-CancerNet model showed the highest accuracy (99.85%), followed by CNN (99.70%) and Vision Transformers (ViT) (99.112%) for early detection of cervical cancer. However, these models performed best on the retrospective dataset. Many such AI tools perform well in controlled settings but struggle in population-level validation. Such models should also be optimized for their integration into the real-world population.

### AI in radiology and imaging for CC diagnosis

The local or distant spread of CC significantly impacts the survival rate of patients. Therefore, a thorough evaluation of the tumour stage at diagnosis is necessary to treat the patient. Imaging is a critical component in deciding the mode of treatment. Commonly used imaging modalities for CC include ultrasound, MRI, Computed Tomography (CT), and positron emission tomography (PET-CT). A growing body of research has highlighted the significance of MRI as the most accurate approach for staging CC (Table 2). Kalantar et al. (2023) used U-Net model, a deep Learning Framework with Multi-Head Dilated Encoders, T_2_-weighted MRI to detect CC from MRI images [47]. The U-Net model is for defined image segmentation and doesn’t require multiple runs to perform image segmentation. Its performance can be enhanced with an increase in the dataset; however, excessive down-sampling can lead to loss of spatial information. The U-Net model gives high accuracy on limited data, with precise segmentation, but its interpretability is poor. MRI is a useful method that provides information on tumour size, vaginal involvement, bladder and rectal infiltration, and detection of metastases to the pelvic and para-aortic lymph nodes.

An MRI-based deep-learning model was developed to predict lymph node metastasis (LNM) in CC patients. The study recruited a total of 392 patients, and the clinical parameters were analyzed using logistical regression to construct a clinical model (M1) and ResNet50 (M2) that extracted the features of the tumour region to develop a multiple-instance MRI-based deep learning hybrid model [48]. Another MRI-based model, ConvNeXt, was used to develop Cer-ConvN3Unet, a multiparametric MRI-based pipeline by employing data of three different MRI sequences of 125 CC patients from two different institutes [49]. This improved the diagnostic performance of the radiologists, which effectively enhanced the clinical decision-making.

**Table 2:** AI-based MRI in CC screening and diagnosis

Model	Study	Model performance	Study outcome	References
U-Net model, deep Learning Framework with Multi-Head Dilated Encoders T _2_ -weighted MRI	Retrospective cohort of 207 patients diagnosed with CC	median Dice similarity coefficient (DSC) score, 0.823 (confidence interval (CI), 0.595–0.797)	Creation of reliable and transferable models for improved CC segmentation on multiparametric MRI	[47]
Deep multiple instance learning (D-MIL) model combined with clinical parameters	Retrospective study	AUC of the training/internal/external cohort of proposed model M3 was 0.838/0.764/0.835	The proposed hybrid model could be used as a personalised non-invasive tool, which is helpful for predicting LNM in operable CC	[48]
ConvNeXt Model	Retrospective study of MRI of 125 CC patients from two different centers	The AUC of the model for each type of MRI slice was between 0.96 to 0.99	This model has increased performance in identification and is more precise and less time-consuming than a gynecologic radiologist	[49]

### Prediction of CC biomarkers for early detection by AI

Prediction of CC biomarkers using AI is an active and promising research area in the field of medicine. Biomarkers are measurable indicators that can help to detect the presence or progression of diseases, including CC. AI algorithms, especially ML, have shown great potential for analyzing large datasets and identifying patterns that humans may find difficult to discern. Biomarker prediction in CC, using AI involve multiple steps, such as collection and preprocessing of data, selection of features and a ML model, model training, model evaluation, biomarker prediction, and final clinical validation. Notably, AI-based biomarker prediction for CC remains an evolving field of research. Scientists and medical professionals are continually working to ameliorate the delicacy, trustworthiness, and clinical connection of AI models for the early detection and substantiated treatment of CC. The success of AI in this sphere depends on the space of high-quality, different, and well-annotated datasets, as well as collaboration between medical experts and AI experimenters. In addition, non-supervisory and ethical considerations are pivotal for ensuring the safe and responsible deployment of AI in healthcare. Owing to its strong data integration and processing skills, AI can screen for specific markers with low consumption and high efficiency, and its benefits are well-illustrated for creating disease-related risk prediction models (Table 3).

A group of researchers used theoretical approaches such as SHapley Additive Extensions (SHAPE) to examine the predicted results by the random forest algorithm model [50]. In another study, ML methods such as neural networks (mmvec) and Random Forest algorithm were employed on integrated multi-omics (microbiome, metabolome, and immunoproteome) datasets to develop a predictive model for identifying genital inflammation and disease status. Random forest algorithm is versatile, has high clarity, is easily comprehensible, and reduces variation, hence works best for the prediction of biomarkers [51].

In another study, big data on DNA methylation was used [52] to identify differentially methylated CpG spots (DMCs) as biomarkers of CC. An AI algorithm was used to study the DNA methylation profile in CC by retrieving the information from the TCGA database. Furthermore, a predictive model integrated with the specific methylated CpG sites was constructed using rbsurv and Cox regression analyses, which significantly predicted the survival of patients. Several epigenetic and demographic features have been reported as predictive biomarkers for CC risk in Indian women. ML-assisted cytokine gene variant analysis and sociodemographic features can be utilized to predict the CC development risk [53]. CC is mostly associated with recurrent high-risk human papillomavirus (hrHPV) infection. Researchers hypothesized that an integrative ML approach could aid in discovering virus-host-associated genetic biomarkers that are important for CC detection or prognosis [54]. Cytoreader-V2, a CNN-based model, was developed to screen CC, which enhanced consistency and reproducibility, but it involves the risk of bias [55].

**Table 3:** AI-driven prediction of biomarkers/prognosis of CC

Model	Study parameters	Model analysis	Model performance	Study outcome	References
Random forest model	Compared the 16 S rRNA sequencing dataset of the vaginal microbiome between 54 healthy and 65 CC individuals	SHAP, a game theoretic approach, is employed to analyze the results predicted by the model.	The prediction probability confidence of the model on the CC and control sample is 0.07 and 0.85, respectively	*Ralstonia* was identified as a microbial predictor marker for CC	[50]
Random Forest	ML approach applied on the dataset that independently analyzed for microbiome, metabolome, and immunoproteome analysis from precancerous and CC patients	Neural networks, Random Forest Classification, Regression	Predicted high genital inflammation with good accuracy (AUC 0.95)	Metabolites primarily long chain fatty acids, sphingolipids, glucose, protein biomarkers interleukin-6 (IL6), interleukin-10 (IL10), and macrophage inflammatory protein 1α (MIP1α) were indicated to be the top predictors of inflammation, a crucial hallmark of CC progression	[51]
Empirical Bayes ML	Data loaded from the IDAT file, DMCs	rbsurv, Cox regression analysis, Kaplan–Meier analysis	CpG biomarker model displayed improved specificity and sensitivity in forecasting the overall survival (AUC = 0:833) of patients with CC	CpG sites could be considered as biomarkers for classifying high-risk or low-risk groups.	[52]
ML based model	With different ML techniques, a dataset comprising cytokine gene variations, clinical and sociodemographic features of healthy normal control people, and CC patients were analysed to identify risk variables.	Accuracy, sensitivity F-1 score and so on	With 84.78% accuracy and 97.83% sensitivity, ridge classifiers exceed the majority of ML classifiers.	ML-assisted investigation of sociodemographic traits and cytokine gene variations to forecast the risk of developing CC	[53]
Integrative ML	Gene expression analysis of datasets obtained from the National Center for Biotechnology Information (NCBI) gene base expression omnibus	NES (Normalized enrichment scores), FDR (false discovery rate) of each gene	Four upregulated genes and five downregulated genes significantly enriched the malignant modules and related genes	Meta-analysis and ML integration effectively recognized the possible genetic markers involved in CC	[54]
Cytoreader-V2	5722 images of dual-stain cytology, using p16 and Ki67 from various studies	Used a convolutional neural network-based classifier and an Ensemble classifier with test-time augmentation	Robustness of Cytoreader-V2 for dual stain positive is 95.0, and dual stain negative is 97.7	High sensitivity and accuracy of Cytoreader-V2 can improve diagnosis	[55]

### AI in drug discovery and therapeutics

AI is a sophisticated approach that has proven useful in identifying novel anticancer targets and innovative therapeutics from multiomics (epigenetics, genomics, proteomics, and metabolomics) technologies. For instance, ML-based biology analysis algorithms can be integrated on multiomics data to identify novel targets for cervical cancer early detection [56]. AI algorithms have been applied to accelerate drug discovery in CC by identifying potential therapeutic targets and predicting the efficacy of existing drugs or novel compounds. Current therapeutic strategies for CC involve artificial intelligence to improve various aspects of cancer treatment, including drug discovery, novel therapies, personalized medicine, and treatment monitoring.

The integration of molecular information in comparative genomics has noticeably improved due to the advanced network-based biological (sequence-similarity genome and gene family networks) analysis. Such approaches first gather expression and interaction data, then transform them into biological procedures for identifying cancer subtypes that can help develop therapeutic targets. Novel therapeutic leads for CC, including proteins (e.g., CRYAB, CDK1, PARP1, WNK1, GSK3B, and KAT2B), metabolites (arachidonic acids), miRNAs, transcription factors (TFs), and receptors, could be found by integrating gene expression profiles into genome-based molecular networks [57].

Integration of AI and nanotechnology can help in the development of advanced personalized therapeutics with reduced or no toxicity. Recently, nano-encapsulated bioactive compounds have been shown to exhibit anticancer activity in CC [58]. However, the toxic properties of these nanoformulations may be difficult to assess in clinical practice. Thus, computational pharmaceutical technology using AI algorithms (Nearest-Neighbor classifiers, RF, extreme learning machines, SVMs, and DNN) can help in determining the toxicity of such novel drugs that will improve the current treatment strategy compared to the traditional way [59].

The application of DNN is being used to predict the different pharmacological properties of multiple drugs as well as drug repurposing using transcriptomics data [60]. Drug repurposing refers to the identification of new indications/uses of already approved or well-established clinical drugs. It reduces turnaround time (TAT) that is otherwise required during conventional development of pharmaceutical drugs. Different levels of predictive tasks can be performed for drug repurposing by using ML and AI models. Drug repurposing predictions use novel drug-disease associations, cell-based composite response predictions of drug-cell line/patient interactions, and biochemical bioactivity predictions of new drug-target interactions [61, 62].

Personalized medicine or precision medicine in cancer involves prescribing a particular medication to a patients based on their tumor heterogeneity, physiology, and lifestyle. Current challenges in cancer therapies include delays in diagnosis and decision-making, imprecise staging, and inconsistent therapy response. An enhanced understanding of the pathogenesis of cancer, tumor stage, and metastatic pathways has increased the development of precision medicine in cancer management. Recent advances in histopathological image analysis and different omics approaches (epigenomics, transcriptomics, proteomics, and metabolomics) have improved personalized therapies in cancer [63]. Currently, 90% of chemotherapies fail due to drug resistance in patients with metastatic cancers, due to tumor heterogeneity. For past twenty years, immunotherapy, especially ICB (immune checkpoint blockade) therapy, has been used effectively for treating metastatic tumors. However, only few patients benefit from this approach, due to the heterogeneity of the disease and acquired resistance during treatment [64]. AI-based analysis of patient big data by using omics technologies (genomics, proteomics, metabolomics) and clinical history of individual patients, can help in the development of personalized treatment [65]. AI can analyze next generation sequencing (NGS) data to identify commonly mutated genes, abnormal expression of genes, and tumor biomarkers [66, 67]. Applying ML to EHRs will help to create personalized medicine by making them reliable risk predictors and accounting for patient variability in disease management and prevention [68–70]. In addition, one study successfully combined peri- and intra-tumoral radiology from highly heterogeneous MRIs (before treatment) to forecast clinical response to neoadjuvant chemotherapy (NACT) in patients with locally advanced cervical cancer (LACC) with excellent accuracy for clinical decision-making [71].

## Advantages, limitations and challenges for AI-based CC screening method

The application of AI for cervical cancer screening and diagnosis has a strong potential for early detection as well as enhanced clinical decision-making. AI can reduce time constraints, limit the number of trained staff and technical experts, improve the specificity and accuracy of CC screening and diagnostic methods, and avoid bias based on subjective components during diagnosis [72]. In areas with limited resources, the incidence of cancer can be significantly reduced by AI-based CC screening. However, despite advantages in AI-assisted cervical cytology screening, its clinical validation and real-world deployment face several challenges [73]. These include high setup costs, reliance on the quality of training data, variability in diagnostics, and complicated approval processes without unified standards. Most studies are retrospective. Standard regulations, multicenter prospective studies, and training for clinicians are essential to ensure balanced technical improvement and clinical use [74]. Further, the use of AI models raises a variety of ethical and societal issues that need to be resolved in order to allow equitable access.

Among the top concerns is the representation and diversity of data sets that will be employed to train AI algorithms. The majority of publicly accessible cervical image databases and cytology data come from high-income countries (HICs), where screening facilities, imaging devices, and patient populations are dissimilar to those in low- and middle-income countries (LMICs). This disparity could lead to the development of AI models that perform poorly on LMIC populations, because of a lack of resources and limited expertise thereby impacting AI-based screening [75]. Algorithmic bias is not limited to the diversity of datasets but also incorporates inherent biases in data annotation, image quality, and labelling standards. In the event that ground-truth labels are taken from inconsistent or subjective expert judgments, the generated AI model can propagate those biases. For instance, models trained largely on data from HIC can show varying results for LHIC. This unevenness might continue current health inequities instead of reducing them. Therefore, there is an urgent need for cross-region collaborations and open-access data availability representative of diverse ethnic groups, clinical environments, and imaging modalities to support the fairness and generalizability of AI systems.

The second most important challenge is data privacy and ethical data governance [75]. Screening for cervical cancer entails intimate personal and reproductive health information, hence the need for informed consent, anonymization of data, and safe storage of data. In most LMICs, digital health legislation is not fully developed, resulting in limited legal protection for patient data. Mobile-based AI screening devices like smartphone colposcopy or cloud-based diagnosis systems also increase the risk of unauthorized data exposure and breaches. Compliance with global standards like the General Data Protection Regulation (GDPR) or their local counterparts, as well as an open data-sharing policy, is necessary to protect the public’s faith and ensure ethical practices.

Lastly, interpretability and regulatory control pose major challenges to clinical translation. Most deep learning models applied to cervical image classification are “black boxes,” providing good accuracy but poor understanding of the decision-making process. This absence of explanation complicates trust in the clinicians and makes accountability in the event of diagnostic failure even more difficult. In addition, no single global framework for the regulation of AI in medical imaging exists today; thus, the approval process varies among regions. There is a need for validation of AI tools in actual clinical environments, ongoing post-deployment monitoring, and open reporting of performance metrics as major steps towards the formation of regulations. Only after solving these ethical, technical, and governance challenges can AI-based cervical cancer detection systems gain safe, equitable, and globally acceptable clinical integration.

## Conclusions

Several AI-based techniques for imaging, radiology or MRI have demonstrated accuracy and precision in predicting cervical cancer. Among them, CNNs, RL-CancerNet and Vision Transformers AI tools using imaging databases have the potential to significantly improve the current cervical cancer screening paradigms. Scientists and medical professionals are continually working to enhance the specificity of AI models for early detection and targeted treatment of CC. However, healthcare professionals should consider the ethical challenges and regulatory hurdles for the translation of AI tools into actual CC screening paradigms. AI-based biomarker prediction for CC is an evolving field. This approach can lead to the development of a potential, portable and low-cost POC detection device that could accelerate the elimination of cervical cancer worldwide, thus supporting multiple sustainable development goals (SDGs).

## Data Availability

No datasets were generated or analysed during the current study.

## Abbreviations

CC: Cervical Cancer
AI: Artificial Intelligence
ML: Machine Learning
Pap: Papanicolaou
CIN: Cervical Intraepithelial Neoplasia
LSIL: Low-Grade Squamous Intraepithelial Lesion
HSIL: High-Grade Squamous Intraepithelial Lesion
ICC: Invasive Cervical Cancer
WHO: World Health Organization
HPV: Human Papillomavirus
VIA: Visual Examination with Acetic Acid
LBC: Liquid-Based Cytology
POC: Point of Care
MRI: Magnetic Resonance Imaging
DL: Deep Learning
DIPS-PCR: Detection of Integrated Papillomavirus Sequence by Polymerase Chain Reaction
NLCs: Nanostructure Lipid Complex
SCC-Ag: Squamous Cell Carcinoma
CA-125: Cancer Antigen-125
CA 19 − 9: Cancer Antigen 19 − 9
CYFRA 21 − 1: Cytokeratin 19 Fragment Antigen 21 − 1
miRNA: Micro RNA
MLP: Multi-layer perception
SVM: Support Vector Machine
KNN: K-nearest Neighbors
ANN: Artificial Neural Network
DNN: Deep Neural Network
EHRs: Electronic Health Records
CNN: Convolutional Neural Network
AVE: Automated Visual Evaluation
HGSO: Hunger Games Search Optimization
IHC: Immunohistochemistry
LIME: Local Interpretable Model-Agnostic Explanations
RL-CancerNet: Reinforcement Learning Cancer Network
ViT-PSO-SVM: Vision Transformer Integrated with Particle Swarm Optimization and Support Vector Machine
IBGCCRF-SPSST: Improved Boykov graph cut-based conditional random fields and superpixel-imposed semantic segmentation
EVA: Enhanced Visual Assessment
AUC: Area Under Curve
c-MLP: Cascaded Multilayer Perceptron
Swin-GA-RF: Swin-genetic algorithm feature selection-random forest
CT: Computed Tomography
PET-CT: Positron Emission Tomography
LNM: Lymph Node Metastasis
DSC: Dice Similarity Coefficient
CI: Confidence Interval
D-MIL: Deep Multiple Instance Learning
SHAPE: SHapley Additive Extensions
CpG: Cytosine-phosphate-Guanine
DMCs: Differentially Methylated CpG spots
hr-HPV: High-risk human papillomavirus
IL-6: Interleukin-6
IL-10: Interleukin-10
MIP-1α: Macrophage Inflammatory Protein-1α
NCBI: National Center for Biotechnology Information
NES: Normalized Enrichment Scores
FDR: False Discovery Rate
TF: Transcription Factor
TAT: Turnaround Time
NGS: Next Generation Sequencing
NACT: Neoadjuvant Chemotherapy
LACC: Locally Advanced Cervical Cancer
HIC: High-Income Countries
LMIC: Low to Middle Income
GDPR: General Data Protection Regulation
SDG: Sustainable Development Goals
